# Evaluation of the Safety and Efficacy of Ormeloxifene, a Selective Estrogen Receptor Modulator and Medroxyprogesterone Acetate in Women with Non-Structural Abnormal Uterine Bleeding: A Randomized Clinical Trial

**DOI:** 10.3390/medicina58111503

**Published:** 2022-10-22

**Authors:** Suhail Ahmad Mir, Rifat Ara, Fiza Amin, Anjum Malik, Laraibah Hamid, Tabasum Ali, Ghulam Nabi Bader, Shahid Ud Din Wani, Mansour Almuqbil, Sultan Alshehri, Abdulhakeem M. Alshehri, Faiyaz Shakeel

**Affiliations:** 1 Department of Pharmaceutical Sciences, University of Kashmir, Hazratbal, Srinagar 190006, India; 2 Department of Obstetrics & Gynaecology, Sher-I-Kashmir Institute of Medical Sciences, Medical College & Hospital, Srinagar 190001, India; 3 Department of Gynaecology and Obstetrics, Lala Ded Hospital, Government Medical College, Srinagar 190006, India; 4 Department of Zoology, University of Kashmir, Hazratbal, Srinagar 190006, India; 5 Department of Clinical Pharmacy, College of Pharmacy, King Saud University, Riyadh 11451, Saudi Arabia; 6 Department of Pharmaceutics, College of Pharmacy, King Saud University, Riyadh 11451, Saudi Arabia; 7 Supply Department, Armed Forces Aeromedical Center, Dhahran 31932, Saudi Arabia

**Keywords:** abnormal uterine bleeding, medroxyprogesterone acetate, ormeloxifene, selective estrogen receptor modulator, antiestrogenic, health related quality of life

## Abstract

*Background and objectives*: Abnormal uterine bleeding is a significant clinical and gynaecological concern that necessitates its safe and effective treatment. The present study aims to compare the cost-effectiveness, safety, efficacy, and health-related quality of life of ormeloxifene with medroxyprogesterone acetate in women with non-structural abnormal uterine bleeding. *Materials and Methods*: A prospective, randomized, single-blinded clinical trial of 367 patients was carried out at a tertiary care hospital for a period of one year from 5 January 2019 to 4 January 2020. Patients were randomized into two groups for administering ormeloxifene and medroxyprogesterone acetate for a 3-month treatment duration and were evaluated by laboratorial investigations like anaemic status, bleeding duration, endometrial thickness, pictorial blood loss assessment chart (PBLAC) score, and patient’s medical and medication history. Health-related quality of life was assessed using short form survey-36 (SF-36) questionnaire scale. Cost-effectiveness was determined on the basis of the three-month treatment regimen. *Results*: The mean duration of bleeding reduced from 16.88 ± 6.46 to 7.76 ± 1.55 in the ormeloxifene group and from 15.91 ± 5.04 to 8.7 ± 1.91 (*p* < 0.001) in the medroxyprogesterone acetate. Similarly, mean haemoglobin increased from 8.56 ± 0.77 to 10.1 ± 0.087 g/dL and from 8.60 ±0.97 to 9.551 ± 0.90 g/dL (*p* < 0.001), and endometrial thickness showed a reduction from 8.52 ± 1.61 mm to 6.92 ± 1.68 mm and from 8.40 ± 2.09 mm to 7.85 ± 2.0 mm (*p* < 0.001) in the ormeloxifene and medroxyprogesterone acetate groups, respectively. PBLAC score reduced from 289.92 ± 42.39 to 128.11 ± 33.10 and from 287.38 ± 40.94 to 123.5 ± 29.57 (*p* < 0.001) in these groups, respectively. Health-related quality of life improved in the ormeloxifene group more than the medroxyprogesterone group, which was evidenced by SF-36 scale parameters (physical function, energy/fatigue and pain) that changed from 24.39, 12.99, 6.25 to 28.95, 18, 9 and from 25.41, 13.6, 7.1 to 27.02, 16, 8.3 in the ormeloxifene and medroxyprogesterone acetate groups, respectively. *Conclusions*: The study concludes that both medroxyprogesterone acetate and ormeloxifene are safe and efficacious in controlling abnormal uterine bleeding, but ormeloxifene was the better of the two in terms of cost effectiveness, reduction in pictorial blood loss assessment score, endometrial thickness, bleeding duration (days), increase in haemoglobin concentration (g/dL) and improvement in the quality of life.

## 1. Introduction

Abnormal uterine bleeding (AUB) is defined as bleeding from the uterine corpus that is abnormal in duration, frequency, volume and regularity [1,2]. AUB and heavy menstrual bleeding (HMB), a subgroup of AUB, are common conditions affecting 14–25% of women of reproductive age [3]. The prevalence varies in each country (in the United States, United Kingdom and African countries, from 3–30%, with higher incidence occurring around menarche and perimenopause). In India, the reported prevalence of AUB is around 17.9% (National Health Portal, NPH)) and in UK, over 800,000 women seek help for AUB annually [4]. From the gynaecological health perspective, menstrual disorders are the most prevalent gynaecological disorders in the United States; around 30% women suffer from heavy menstrual bleeding (HMB) at some time during their reproductive years [5]. It has a significant impact on both economic and health care costs, directly affecting women and their families. A study carried out by Frick and colleagues [6] claimed losses of more than USD 2000 per patient per annum due to the absence of women from work. In UK gynaecological services, AUB is the fourth most common referral cause. In a recent England and Wales audit report by Royal College of Obstetrics and Gynecology (RCOG), it was highlighted that only a third of women (including those managed by surgery), post 1-year referral were satisfied with the prospect of their current menstrual symptoms continuing, as currently experienced, for the next 5 years [7]. The causes of AUB can be either single or multiple and include structural causes (polyp, adenomyosis, leiomyoma, malignancy/hyperplasia) and non-structural causes (coagulopathy, ovulatory dysfunction, endometrial, iatrogenic, and not otherwise classified), better known as PALM-COEIN [2,8]. Though mortality from AUB is extremely low, its significance lies in its effect on physical, social, and emotional quality of life (QoL), aside from the tremendous impacts on health and productivity loss [9]. Women with menstrual-related problems are more likely to report insomnia, excessive sleepiness, anxiety, depression, and pain [10]. Studies carried out in the United Kingdom and United States have reported that women with AUB show anxiety regarding potential embarrassment and the unpredictability of changing plans (work, family, and social) to avoid an embarrassing bleeding episode that ultimately leads to a reduction in QoL [11,12,13].

The high prevalence of iron deficiency anaemia in women in the developing world has been linked to various factors including poor nutrition and limited or lack of access to simple iron replacement therapies [12]. Women suffer from the adverse effects of AUB in their prime youth. The suffering is only relieved temporarily during pregnancy and lactation and then permanently at menopause. Confusion and inconsistent nomenclature together with lack of standardized methods of investigation and characterization of various potential aetiologies have hampered the investigation and management of AUB among non-gravid women of reproductive age [13]. These factors make the comparison of different studies by different researchers difficult. Keeping all this in mind, a universally accepted nomenclature system PALM-COEIN system of classification was developed and approved throughout the world by International Federation of Gynecology and Obstetrics (FIGO) Executive Board. This system of classification is known as FIGO [2,14]. The most important diagnostic laboratorial tests for AUB include the exclusion of pregnancy (which is confirmed by urine pregnancy test or checking serum β-human chorionic gonadotropin (β-hCG level), coagulation profile (performed to exclude bleeding disorders), and complete blood count (CBC, which is performed to exclude anaemia in case of excessive/persistent or longer duration bleeding episodes). For the evaluation of anovulation, and hormonal disorders like thyroid and prolactin disorders, other laboratorial tests are performed. In polycystic ovarian syndrome and congenital adrenal hyperplasia, usually there are high levels of androgens and 17-alpha hydroxyprogesterone [15]. In addition to ultrasonography, pictorial blood loss assessment chart score (PBLAC), endometrial biopsy, hysteroscopy, and dilatation and curettage are the other diagnostic measures for abnormal uterine bleeding [16]. There is a worldwide lack of consensus among clinicians with regard to medical management in various types of bleeding and the right time to go for definitive surgical intervention. The management approach of AUB is to control the bleeding and then ensure general well-being and improve the QoL. The first therapeutic option for AUB has always been medicine, and in case of failure by medical management, surgical interventions are required. As the last resort for the management of AUB, hysterectomy should be considered. Treatment options available include non-steroidal anti-inflammatory drugs (NSAIDs), antifibrinolytics, levonorgestrel intrauterine system (LNG-IUS), daily hormonal pills, and selective estrogen receptor modulators (SERMs). Though drugs like tranexamic acid, mefenamic acid, flurbiprofen, norethisterone, and medroxyprogesterone acetate (MPA) can decrease the menstrual blood loss by around 50%, yet many women remain menorrhagic, and many are noncompliant due to daily dosing. Thus, the search for an ideal therapy for abnormal uterine bleeding that prevents, bone loss, has a positive effect on the cardiovascular system, and poses no risk of breast or uterine cancer is still going on. SERMs in general and ormeloxifene (ORM) in particular fit this profile. ORM, a non-steroidal drug, is easier to administer and cost effective, and it has fewer side effects. However, there are limited data available with respect to its use in gynaecological diseases, and currently it is restricted to AUB and advocated by very few researchers [3,17]. In this regard, the present study was undertaken to evaluate and compare two drugs, ORM and MPA, for their safety, efficacy, and improvement in health-related QoL (HRQoL) in women suffering from abnormal uterine bleeding with a non-structural cause.

## 2. Materials and Methods

### 2.1. Clinical Trial

A prospective, randomized single-blinded clinical trial was carried out for a period of one year from 5 January 2019 to 4 January 2020 at Department of Obstetrics and Gynaecology, Sher-I-Kashmir Institute of Medical Sciences (SKIMS), Medical College and Hospital, Bemina, Srinagar, a tertiary care hospital that provides specialist care for all age groups.

### 2.2. Clinical Trial Registration

The trial was registered in the Clinical Trial Registry of the India-Indian Council of Medical Research (CTRI-ICMR) Vide Registration number CTRI/2019/01/017230.

### 2.3. Inclusion Criteria

All patients attending the outpatient department (OPD) or arriving for consultation in the obstetrics and gynaecology ward for abnormal uterine bleeding were enrolled (367), although only those having a non-structural cause of abnormal uterine bleeding (240 patients) were included in the study.

### 2.4. Exclusion Criteria

Patients with structural causes like leiomyoma, adenomyosis, polyp, adnexal mass, systemic disorder, breast malignancy, and endometrial hyperplasia were excluded from the study.

### 2.5. Sample Size

After statistical confirmation, out of 367 patients, 240 patients were selected for the study on the basis of the inclusion criteria. Patients were divided into two groups by randomization (simple randomization technique), 120 in each group. Group A (ORM group) was prescribed 60 mg ORM orally twice a week for the initial two months, which was then followed by once a week for one month. Group B (MPA group) was prescribed 10 mg MPA twice daily, starting from day 5 up to day 25 of the menstrual cycle. The treatment continued in the same way for three months. Sample size was calculated using confidence interval of 95%, margin of error 5%, and population proportion of 19%.

### 2.6. Study Population

Female patients in the age group 20–55 years with abnormal uterine bleeding attending OPD at the obstetrics and gynaecology department were enrolled in the study.

### 2.7. Data Collection

**Figure 1:**
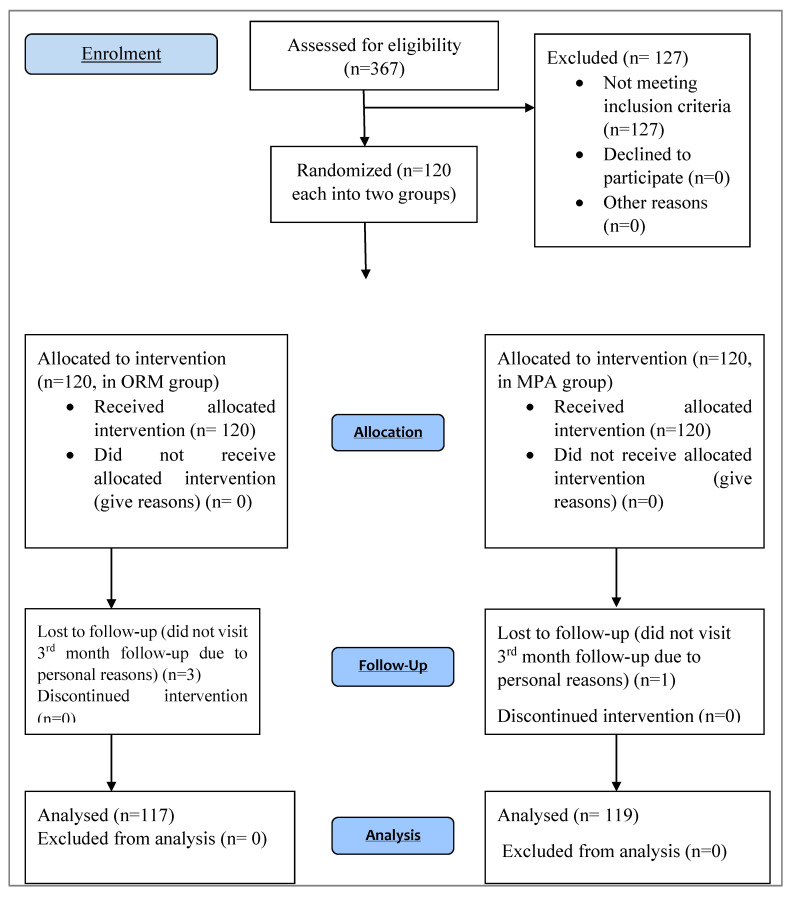
CONSORT diagram of the study.

Data were collected using a pre-structured data collection form. The important data variables included medical record department (MRD) number (for follow up), patient anthropometric details, comorbidities, investigation reports, and medication history. Baseline parameters like PBLAC (a simple and inexpensive tool comprising a visual scoring system that depicts a graded series of soiled tampons and/or towels) [18], haemoglobin value, endometrial thickness, and other related and relevant parameters were collected before starting the therapy. Ultrasonography (USG), hysteroscopy, and dilatation and curettage were for the histopathological evaluation of the endometrium. The parameters were recollected and recorded after the 1st, 2nd, and 3rd months during follow-up visits. A total of 367 women were screened for abnormal uterine bleeding, as shown in Figure 1.

### 2.8. HRQoL (SF-36 Quality of Life Scale)

Data were collected via face-to-face and phone interviews with a questionnaire form validated by the Institutional Ethics Committee in light of the related literature and the SF-36 Quality of Life Scale. The questionnaire consisted of 36 questions regarding women’s sociodemographic (age, education, occupation) features, obstetrics (pregnancy and number of births) and menstruation characteristics, and gynaecological and medical problems. The SF-36 questionnaire consisted of 35 items covering 8 distinct health status concepts (physical functioning, physical role functioning, pain, general health, energy/fatigue, social role functioning, emotional role functioning, and mental health) and one item measuring self-reported health transition. Higher scores indicate greater QoL [19]. The HRQoL was assessed in patients before and after MPA and ORM therapy.

Ormeloxifene treatment was found to be cost effective of the two and had better patient compliance and adherence than medroxyprogesterone acetate in our study. MPA for the 3-month treatment duration costed INR 780, while ORM treatment costed INR 390 (Table 1).

### 2.9. Statistical Analysis

Data were analysed by Statistical Package for Social Sciences (SPSS) software version 20. Descriptive statistics in the form of frequency distribution and associated percentage were employed for the presentation of demographic details. Comparison between the laboratorial values of two drugs was analysed by Student’s *t*-test between the two groups and paired *t*-test (from baseline to 3rd month) within each group with confidence interval of 95%. *p*-values at <0.05 were taken as significant.

## 3. Results

The demographic data like age, body mass index, and other parameters were comparable among the two groups (Table 2). The clinical characteristics of both groups (ORM and MPA) are presented in Table 2 and Table 3. The groups were similar with respect to the baseline clinical and laboratory characteristics at the time of enrolment. Baseline Hb, endometrial thickness, PBLAC score, and bleeding duration levels in the two groups were clinically insignificant at *p* > 0.05 (Table 2).

**Figure 2:**
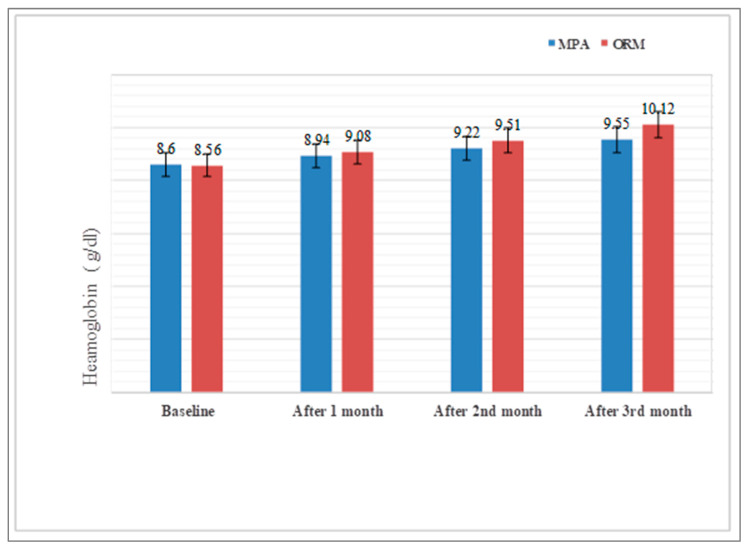
Comparison of haemoglobin g/dL levels before and after MPA and ORM therapy. Bars represent significance.

**Figure 3:**
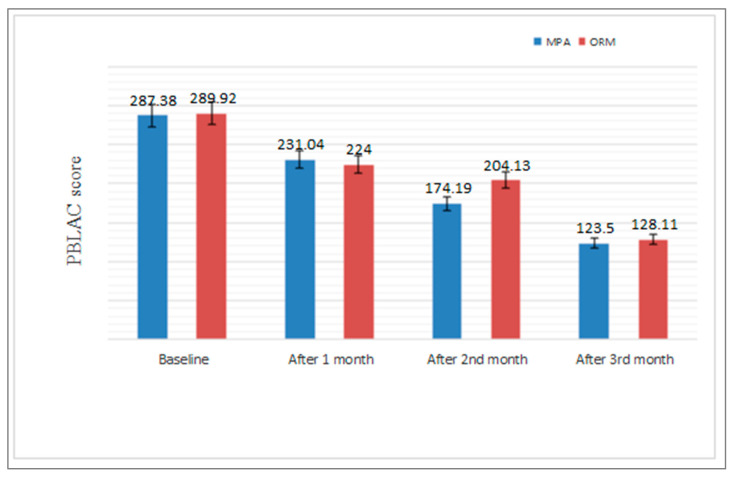
Comparison of PBLAC score before and after MPA and ORM therapy. Bars represent significance.

**Figure 4:**
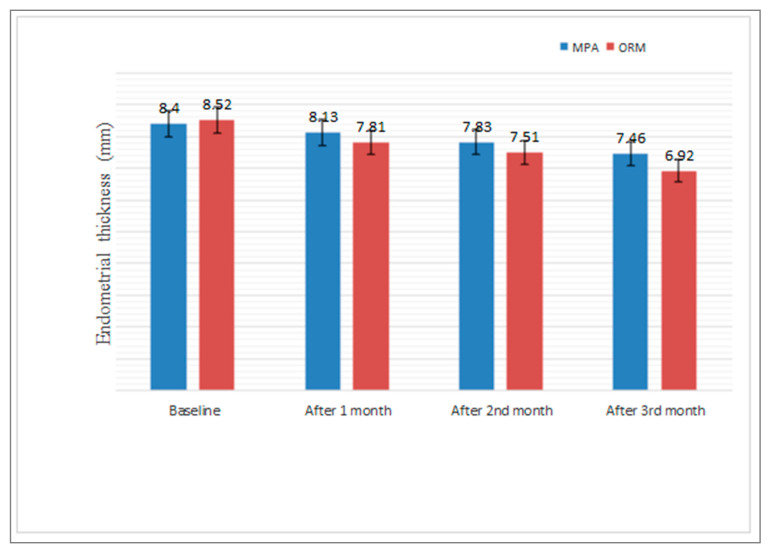
Comparison of Endometrial thickness (mm) levels before and after MPA and ORM therapy, Bars represent significance.

Regarding measures of efficacy, the patients who received ORM in the 3rd month of the treatment reported a significant improvement with respect to Hb and a reduction in endometrial thickness, PBLAC score, and bleeding days than patients that received MPA (*p* < 0.0001 for Hb, endometrial thickness, and PBLAC and *p* < 0.024 for bleeding days) (Table 3 and Figure 2, Figure 3 and Figure 4),when compared with baseline characteristic with respect to Hb value(*p*-value, 0.809), endometrial thickness(*p* value, 0.809), PBLAC score (*p*-value, 0.964) and bleeding duration (*p*-value, 0.860). There was significant improvement with respect to Hb and reductions in endometrial thickness, PBLAC score from baseline at 1st, 2nd and 3rd months, which was statistically significant within the group at *p* <0.05 (Figure 2, Figure 3 and Figure 4).

It was found that 65.5% of AUB participants had two or more children (Table 2). Histopathological evaluation showed that the endometrium was in proliferative phase in 49.16%, secretory in 20%, hyperplastic in 15.83%, and atrophic in 10.83% and endometritis in 4.18% patients. The age distribution of AUB in the study was 40.4% in 5th decade (41–50 years) and 46.66% of cases were menorrhagic (Table 2).

HRQoL improved in ORM group better than MPA group which was evidenced by SF-36 scale parameters (physical function, energy/fatigue and pain) that changed from 24.39, 12.99, 6.25 to 28.95, 18, 9 respectively in ORM group and from 25.41, 13.6, 7.1 to 27.02, 16, 8.3 respectively in MPA group (*p*-value < 0.0001 within group and *p* < 0.05 between the two groups) (Table 4 and Table 5).

No major side effects (SEs) were seen with use of ORM during the course of study, however, some minor SEs were fever (3%), headache (1%), weight gain (2%), and abdominal cramps (3%) which did not required medical attention and discontinuation of the treatment. Some of the side effects with use of MPA were fever (2.5%), abdominal cramps (3.76%) and headache (3%), which also did not require medical attention. Only 4 (3.3%) patients from ORM group and 7 (5.8%) patients from MPA group opted for hysterectomy owing to dissatisfaction with the drug treatment.

Out of 120 patients that were enrolled for the study of ORM, 3 (2.5%) did not complete the 3rd month follow up;{1 withdrew the therapy and 2 reported oligomenorrhea as an adverse reaction. Similarly, in the enrolled MPA group, 1 (0.83%)} did not complete the 3rd month follow-up due to personal reasons.

## 4. Discussion

The significance of AUB lies in its deleterious effect on physical, social and quality of life, and economic losses like health and productivity loss, although the disease poses extremely low mortality threat. Ormeloxifene treatment was found to be cost effective, a major factor in the developing world for patient compliance and adherence to treatment protocols. On the basis of acceptability and efficacy, our study found that ORM was the preferred drug as dissatisfied with ORM treatment, only 4 (3.3%) patients opted for hysterectomy Whereas, the percentage went up to 5.8% (7 patients) in patients that received MPA. The age distribution of AUB in the study was 40.4% in 5th decade (41–50 years). This finding is in line with some earlier studies. In a study conducted by Saraswathi and co-workers [20], it was found that 33.5% of cases belonged to 41–50 years. Similarly, the study conducted by Abdulla and colleagues [21] concluded that 33.1% cases of AUB were in the 5th decade. The studies conducted by Bindroo et al. and Jairajpuri et al. [22,23] also reported 35.89% and 35.9% cases belonged to 5th decade respectively. The reason for increased incidence of abnormal uterine bleeding in this age group (41–50 years) may be owing to their being in climacteric period. When these women approach menopause, their cycles shorten and often they become intermittently anovulatory due to decreased number of ovarian follicles and their increased resistance to gonadotrophic stimulation, which causes a decline in oestradiol level, which cannot keep the normal endometrium growing. In our study, we found that 46.66% of cases were menorrhagic which is almost in line with the studies conducted by Nayak, Mishra, Rifat and co-workers [24,25,26] which reported 46.66%, 48.8% and 47.2% of menorrhagic patients respectively. The second common pattern of bleeding in our study is metrorrhagia that accounted for 27.5% of cases. This is also almost similar to that found in the studies conducted by Afghan S and co-workers, Mahapatra M and Co-workers [25,27] who reported 20.6% and 23% of cases with metrorrhagia respectively. The reason behind this may be that the most cases of AUB are secondary to anovulation. Without ovulation, the corpus luteum fails to form, resulting in failure of progesterone secretion. Thus, unopposed oestrogen allows the endometrium to proliferate and thicken. The endometrium finally outgrows its blood supply and degenerates resulting in asynchronous breakdown of the endometrial lining.

Histopathological evaluation revealed proliferative endometrium in 49.16% and secretory endometrium in 20% of cases in our study. Earlier studies by Zawar and colleagues [28] reported 43% cases of proliferative endometrium. Doddamani and co-workers [29] observed proliferative endometrium in 44.7% and secretory in 23.5% of cases which is almost similar to our study. Histopathological examination of the endometrium also revealed that whatever may be the pathology, proliferative endometrium is the most common pattern. Shravage and colleagues [30] in a double blind randomized controlled trial on ORM and MPA in dysfunctional uterine bleeding observed a reduction of mean PBLAC score by 85.71% and 54.76% respectively with ORM and MPA after 4 months of treatment. In our study the reduction of mean PBLAC score was 80.4% and 75% respectively.

In a study conducted by Dhananjay and colleagues [31], there was a statistically significant increase in haemoglobin concentration (8.26 to 10.59 g/dL; *p* < 0.001) and a statically similar decrease in the endometrial thickness (8.36 to 4.89 mm; *p* < 0.001) after 3 months of treatment with ORM. In our study, we observed that mean Hb increased from 8.56 ± 0.77 to 10.12 ± 0.087 g/dL in ORM treated group and from 8.60 ± 0.97 to 9.55 ± 0.90 g/dL in MPA treated group; endometrial thickness was reduced from 8.52 ± 1.61 mm to 6.92 ± 1.68 mm in ORM treated group and from 8.40 ± 2.09 mm to 7.85 ± 2.0 mm in MPA treated group. Ravi babu and coworkers [32] in their study found that the mean endometrial thickness with ORM was reduced from 11.67 mm to 9.3 mm and mean Hb increased to 11.2 g/dL from 10.6 g/dL after 4 months of treatment. Our study shows a significant increase in Hb level (*p* < 0.0001) and decrease in endometrial thickness (*p* < 0.0001) which is suggestive of the fact that since ormeloxifene has anti-proliferative effect on endometrium. It causes endometrial atrophy, thereby leading to decrease in menstrual blood loss. Mechanism behind improvement of Hb level and menorrhagia may be due to antagonistic effect of ORM on endometrial oestrogen receptors which enhance prolonged depletion of oestrogen due to decrease in receptor stimulation [33]. Godha Z and colleagues found a reduction in mean duration of bleeding to 4.8 from 9 in ORM and to 5 from 8.7 in MPA group, and mean haemoglobin increased from 8.6 to 9.8 g % and from 8.7 to 9.9 g % in ORM and MPA group respectively, which is in consistence with our study [34]. Priyanka and colleagues in their study showed an increase in Hb concentration and a reduction in mean endometrial thickness, which is also in accordance with our study [35]. Our study also revealed that HRQoL improved more in patients receiving ORM compared with MPA. There was better improvement in physical, social, mental, and general health of patients receiving ORM therapy than MPA. The reason for this may be that ORM has better patient acceptability and compliance due to its minimal side effects, low cost (compared with all alternative medical and surgical treatments), and simple dosage schedule than MPA. To sum up, both MPA and ORM are safe and efficacious. However, ORM in our study showed a more significant reduction in PBLAC score, endometrial thickness, and bleeding duration (days) and greater increases in Hb concentration (g/dL) and HRQoL than MPA.

### 4.1. Strength of the Study

This is the only registered study under the clinical trial registry of India, Indian council of medical research (CTRI-ICMR) for comparison of two oral preparations for AUB in India. It is also the first study on these two drugs related to evaluations of HRQoL. This study adds to the very little existing data with respect to the comparison of safety, efficacy, and HRQoL of MPA and ORM.

### 4.2. Limitations of the Study

Small sample size, short duration, and single-centre trial are the limitations of this study. A large multicentre, randomized controlled trial is required for further validation the results.

## 5. Conclusions

AUB is an ever-increasing gynaecological disease, having impacts on patients’ physical and mental health in addition to economic implications. The approach to management should ensure general well-being and improved quality of life. Our study concludes that both MPA and ORM are safe and efficacious in this regard. However, ORM is better of the two as it reduced PBLAC score, endometrial thickness, bleeding duration (days), and increased Hb concentration (g/dL) and improved HRQoL better when compared with MPA. ORM controlled AUB without effecting normal endocrinal and physiological parameters and without any major side effects.

## Figures and Tables

**Table 1:** Showing the total expenditure on complete dose regimen of two drugs.

Particulars (Drug Used)	Trade Name and Manufacturer	Total Dose/Tablets Needed	Price/Strip of 10 Tablets (INR)	Total Expenditure for 3 Months (INR)
Medroxyprogesterone Acetate	Deviry Torrent pharmaceuticals	12.6 strips of 10 tablets (13 strips)	60	780
Ormeloxifene	Sevista Torrent pharmaceuticals	2 strips of 10 tablets	195	390

**Table 2:** Baseline clinical and laboratory characteristics at the time of enrolment. Demographic data like age, body mass index, and other parameters were comparable among groups.

Baseline Demographic and Clinical Characteristics of the Study Patients			
Variable	Ormeloxifene (n = 120)	Medroxyprogesterone Acetate (n = 120)	*p*-Value *
Age-years	40.31 ± 5.852	39.90 ± 5.418	0.926
Height—m	1.659 ± 0.107	1.694 ± 0.129	0.585
Body Weight—kg	66.60 ± 6.054	66.72 ± 6.872	0.928
Body Mass Index (BMI^#^^)^—kg/m^2^	23.37 ± 3.70	23.63 ± 4.574	0.884
Comorbidities			
Diabetes	22	33	
Sub-Clinical Hypothyroidism	19	24	
Urinary tract infection	12	8	
Obesity	43	34	
Laboratory Parameters before the start of therapy			
Haemoglobin—g/dL	8.56 ± 0.77	8.60 ± 0.97	0.809
PBLAC Score	289.92 ± 42.39	287.38 ± 40.94	0.964
Endometrial Thickness (mm)	8.52 ± 1.61	8.40 ± 2.09	0.808
Bleeding Duration (Days)	16.88 ± 6.46	15.91 ± 5.04	0.860
Relationship between parity and Abnormal uterine bleeding found in this study (Percentage is calculated as cases divided by total number of cases in both the groups)			
Parity	No of patients (%)	No of patients (%)	
Unmarried	14 (5.83%)	10 (4.16%)	
Nullipara	25 (10.41%)	33 (13.75%)	
Parity 2	23 (9.58%)	35 (14.58%)	
Multiparity	58 (24.16%)	42 (17.5%)	
Distribution of AUB cases according to age (Percentage is calculated as cases divided by total number of cases in both the groups)			
Age Group In years	No of patients (%)	No of patients (%)	
20–25	0 (0%)	0 (0%)	
26–30	9 (3.75%)	13 (5.41%)	
31–35	20 (8.33%)	24 (10%)	
36–40	37 (15.41%)	34 (14.16%)	
41–45	50 (20.83%)	40 (16.66%)	
46–50	2 (0.83%)	5 (2.08%)	
51–55	2 (0.83%)	4 (1.66%)	
Incidence of various modes of presentation of abnormal uterine bleeding (Percentage is calculated as cases divided by total number of cases in both the groups)			
Type	No. of patients (%)	No. of patients (%)	
Menorrhagia	60 (25%)	52 (21.66%)	
Metrorrhagia	31 (12.91%)	35 (14.58%)	
Polymenorrhagia	21 (8.75%)	19 (7.91%)	
Polymenorrhoea	5 (2.08%)	9 (3.75%)	
Continuous bleeding	3 (1.25%)	5 (2.08%)	

* *p*-values calculated by students-test; mean ± standard deviation; ^#^, body mass index is the weight in kilograms divided by height in meters.

**Table 3:** Baseline and 3rd month clinical data of ormeloxifene and medroxyprogesterone acetate.

Baseline Clinical Data			
Laboratory Parameters	Ormeloxifene (n = 120)	Medroxyprogesterone Acetate (n = 120)	* *p*-Value
Haemoglobin—g/dL	8.56 ± 0.77	8.60 ± 0.97	0.809
PBLAC Score	289.92 ± 42.39	287.38 ± 40.94	0.964
Endometrial Thickness (mm)	8.52 ± 1.61	8.40 ± 2.09	0.808
Bleeding Duration (Days)	16.88 ± 6.46	15.91 ± 5.04	0.860
**3rd month clinical data**			
**Laboratory Parameters**	**Ormeloxifene** **(n = 117)**	**Medroxyprogesterone Acetate** **(n = 119)**	* *p*-value
Haemoglobin—g/dL	10.12 ± 0.87	9.55 ± 0.90	0.0001
PBLAC Score	128.11 ± 33.10	123.5 ± 29.57	0.0001
Endometrial Thickness (mm)	6.92 ± 1.68	7.46 ± 1.97	0.0001
Bleeding Duration (Days)	7.76 ± 1.55	8.7 ± 1.91	0.024
* *p*-value (within the group)	0.0001	0.0001	

* *p*-values are based on paired *t*-test within the two groups and student’s *t*-test between the two groups.

**Table 4:** Comparative findings about SF-36 quality of life scale after ORM and MPA therapy.

Dimensions of Quality of Life Scale	Group A (ORM) Before Therapy	Group A (ORM) After Therapy	Group B (MPA) Before Therapy	Group B (MPA) After Therapy	*p*-Value (Between the Groups)	*p*-Value (Within the Groups)
Physical function	24.39	28.95	25.41	27.02	0.0001	0.0001
Social functioning	7.26	9.2	8.1	8.6		
Mental health	17.21	20.1	16.95	18.2		
General health	15.35	18.32	15.9	16.3		
Role physical	1.72	2.9	1.6	2.2		
Role emotional	1.49	3.2	1.83	2.63		
Energy/Fatigue	12.99	18	13.6	16		
Pain	6.25	9	7.1	8.3		

**Table 5:** Comparison of ORM and MPA Group on level of health and functioning subscale among the patients with abnormal uterine bleeding after therapy.

Health and Functions	Group A (ORM) (%)	Group B (MPA) (%)	*p*-Value
Very dissatisfied	5	10	0.0001
Moderately dissatisfied	10	20	0.0001
Slightly dissatisfied	20	31.66	0.0001
Slightly satisfied	31.66	16.66	0.0001
moderately satisfied	16.68	11.68	0.0001
Very satisfied	16.66	10	0.0001

## Data Availability

Not applicable.
